# Extent and nature of dual practice engagement among Iran medical specialists

**DOI:** 10.1186/s12960-018-0326-4

**Published:** 2018-11-20

**Authors:** Mahboubeh Bayat, Gholamhossein Salehi Zalani, Iraj Harirchi, Azad Shokri, Elmira Mirbahaeddin, Roghayeh Khalilnezhad, Mahmoud Khodadost, Mehdi Yaseri, Ebrahim Jaafaripooyan, Ali Akbari-Sari

**Affiliations:** 1 Center for Health Human Resources Research & Studies, Ministry of Health and Medical Education, Tehran, Islamic Republic of Iran; 2 Department of Surgery, School of Medicine, Tehran University of Medical Sciences, Tehran, Islamic Republic of Iran; 3 Social Determinants of Health Research Center, Kurdistan University of Medical Sciences, Sanandaj, Islamic Republic of Iran; 4 Telfer School of Management, University of Ottawa, Ontario, Canada; 5 Health Management and Economics Research Center, Iran University of Medical Sciences, Tehran, Islamic Republic of Iran; 6 Department of Epidemiology, School of Public Health, Shahid Beheshti University of Medical Sciences, Tehran, Islamic Republic of Iran; 7 Department of Epidemiology, School of Public Health, Iran University of Medical Sciences, Tehran, Islamic Republic of Iran; 8 Department of Epidemiology, School of Public Health, Tehran University of Medical Sciences, Tehran, Islamic Republic of Iran; 9 Department of Health Management and Economics, School of Public Health, Tehran University of Medical Sciences, Poursina St, 16 Azar St, Bolvar Keshavarz, Tehran, Islamic Republic of Iran

**Keywords:** Medical specialists, Dual practice, Multiple jobs holding, Iran

## Abstract

**Background:**

Dual practice (DP) by medical specialists is a widespread issue across health systems. This study aims to determine the level of DP engagement among Iran’s specialists.

**Methods:**

A pre-structured form was developed to collect the data about medical specialists worked in all 925 Iran hospitals in 2016. The forms were sent to the hospitals via medical universities in each province. The data were merged at the national level and matched using medical council ID codes, national ID codes, and eventually a combination of the first name, surname, and father’s name.

**Results:**

A total of 48 345 records were collected for 30 273 specialists from 858 (93%) hospitals out of total 925 hospitals. Sixteen thousand eight hundred forty-nine (69% of) specialists were non-faculty members and 6317 (26% of) specialists were employed on a contract basis. Eleven thousand six hundred and thirty-eight (47.7% of) specialists were engaged in DP on total. Female specialists had 0.78 times less DP chance; faculties compared to non-faculties had 0.65 times more DP chance and full-time geographic specialists compared to non-full-time specialists had 0.15 times more DP chance. DP was more frequent in specialists with higher age and more job experience and in provinces with more population, deprivation, and higher number of specialists per facility (*P* < 0.05).

**Conclusions:**

The level of DP is relatively high among Iran medical specialists, especially in geographic full-time specialists. However, they are totally banned and they receive extra payment for being full-time; restrictive regulations and financial incentives without considering other factors might not eliminate DP in specialists and it should be addressed based on conditions of each country and regions inside the country.

## Background

Dual practice (DP) or multiple jobs holding is considered as holding a job in more than one facility simultaneously. DP normally happens when a professional simultaneously works in public and private facilities, in two public facilities or in two private facilities [1, 2]. Kiwanuka et al. define DP for healthcare professionals as having more than one medical job inside or outside public sector facilities [3]. Dual practice is relatively frequent in healthcare, especially in physicians, nurses, midwives, and technicians. However, it seems that specialists have a greater tendency for DP [3–5].

In Austria, 100% of specialists work in both public and private sectors at the same time, followed by 90% in Ireland, 80% in Bangladesh, 71% in Egypt, 70% in Indonesia, 65.7% in Australia, 61% in the UK, 43% in Portugal, 38% in New Zealand, and 37% in Denmark, and income increase is reported as the main reason for DP [1, 2, 4, 6–9].

The income gap between the specialists in the public and private sectors is the main reason to encourage physicians to leave the public sector or work simultaneously in the public and private sectors. Other driving reasons for this flow and multiple job holding can be factors such as lower chance of career development in the public sector, higher autonomy in the private sector, and insufficient infrastructure in public facilities [8].

DP has negative and positive effects in developing and developed countries; however, the negative effects far exceed the positive [4, 11]. The positive consequence was the additional income for health workers that may have resulted in increasing the retention of skilled workers especially in deprived areas and decreasing the budgetary burden of the public sector. Among the negative consequences, this phenomenon has resulted in fragmentation of specialists’ supply and decreased accessibility in the public sector, especially considering the division of specialists’ working time in different locations [10]. Consequently, DP reduces hours of physicians’ presence, imposes more pressure on nurses and other health professionals, and has negative impacts on the quantity and quality of services [1, 2, 11]. It also increases patient referrals from the public to private sectors which can lead to induced demand and increased healthcare costs [12]. Besides, the physicians who are involved in DP might become free riders of the public sector using equipment and resources or administrative services, and nurses of the public sector for private patients [2, 6, 13].

The income gap between the public and private sectors in these countries is a key motivating factor for physicians to leave the public sector or work in both the public and private sectors [8]. Other factors that have been identified as driving the movement of physicians from the public to the private sector include lack of academic and career development opportunities in the public sector, poor infrastructure in public facilities, and greater autonomy in the private sector [8]. Bearing in mind the considerable negative impacts of DP, health systems in different countries address this issue differently based on their context and level of development. Developed countries normally apply partial restrictions through regulatory mechanisms while developing countries use some restricting and mandatory mechanisms [14, 15]. However, these strategies may not always work. Efforts to ban DP in Greece from 1983 to 2002 failed due to lack of capacity to enforce it [10]. In some states of India where DP has been banned, weak enforcement and poor mechanisms to check the practice lead to failure in such obligations [16].

The current strategies used to control DP in Iran include complete ban for full-time geographic (FTG) specialists [17] and partial restrictions using different incentives for non-full-time specialists [18]. FTG physicians are the ones who are not allowed to be active in any other locations/sectors except their main occupation location. The first strategy refers to specialist physicians who are supposed to be full-time (54 h per week), and according to this law, physicians will receive remuneration for full-time status, which will eventually earn more than other doctors [17]. The latter one is related to other experienced non-full-time specialists who are motivated to work full-time in the public sector with no practice in the private sector through some benefits such as paying for having no office equal to their salaries and remuneration for full-time status for non-faculty members, about 60–65% of the salary, under the condition of permanent employment in the public sector (non-DP( [18].While each of these strategies might have different impacts on the workforce and health status in terms of appropriate management and control of DP, countries should design and adopt proper strategies according to their situations, and the first step for the adoption of effective strategies is to identify and explore the level, nature, and contributory factors of DP [1, 19]. In Iran, there is limited evidence about the extent and nature of DP and its contributory factors [14, 20]. This study aims to determine the level of DP engagement and its contributory factors among Iran’s specialists.

## Methods

Since there was no comprehensive data bank regarding medical specialists to detect their behaviors such as DP, we designed a data collection form to create a data bank and extracted physicians with DP through data matching and detecting duplicate data. Some studies from different countries were done through sampling and questionnaire [4, 8–10]. We used this method as a novel method for decreasing the information bias.

### Data collection process

A pre-structured form together with a manual about how to complete it were developed to collect the data about specialists who worked in Iran public and private hospitals in 2016. Study hospitals were all 925 Iranian hospitals including government teaching hospitals; hospitals from the Social Security Organization (SSO), armed forces, and the Ministry of Petroleum (MoP); and other public, private, and charity hospitals.

**Fig. 1:**
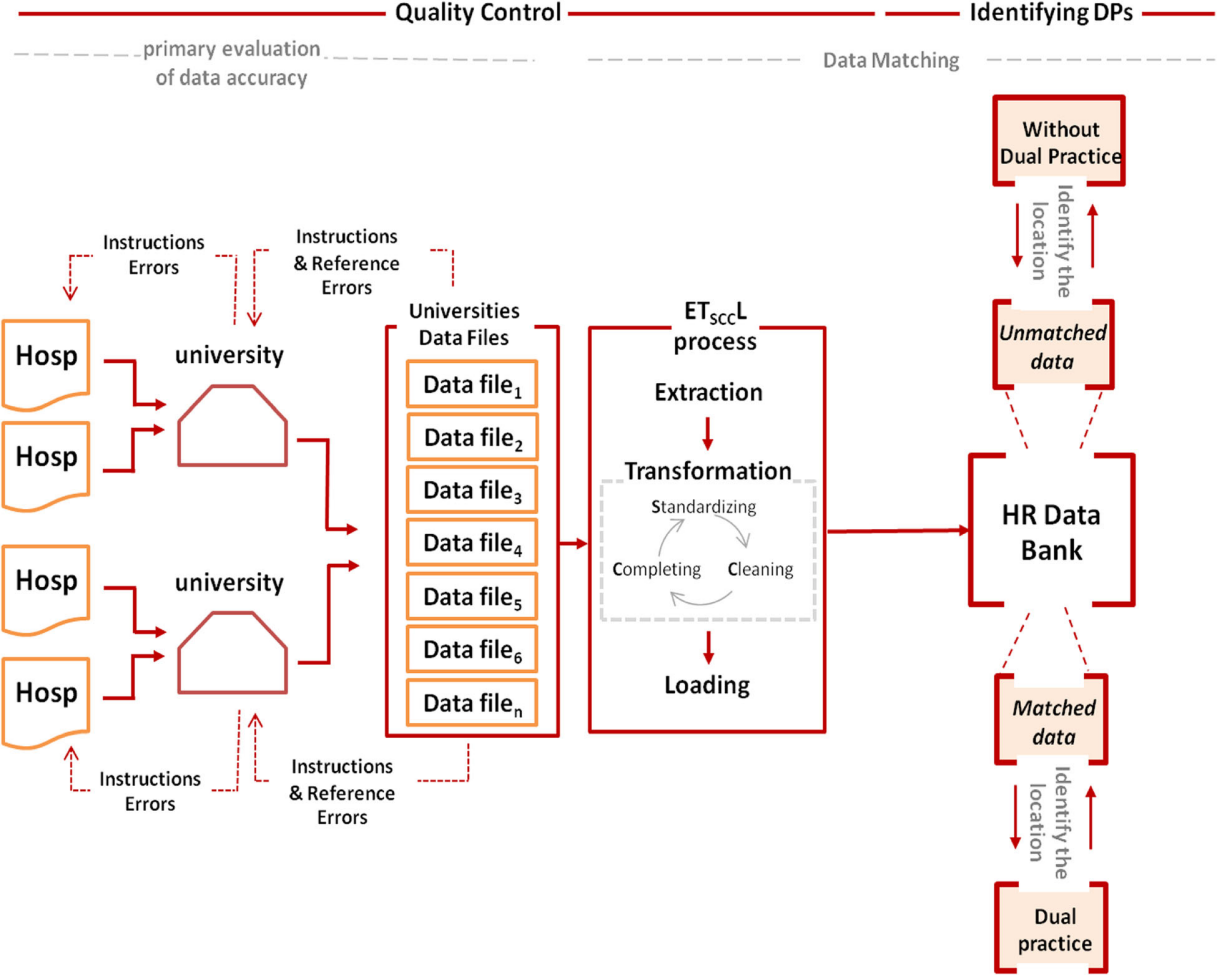
Data extraction process to identify medical specialists’ DP engagement

In each province of Iran, there is at least a government medical university directed by the Ministry of Health and Medical Education (MOHME) that is responsible for education (training of medical doctors, specialists, nurses, etc.) and health service delivery. These medical universities have their own healthcare facilities but at the same time they supervise all other public and private healthcare facilities in their catchment areas. Therefore, the forms were sent to all medical universities via MOHME and the medical universities sent the forms to all hospitals in their catchment areas. Medical universities then collected the data from hospitals of their catchment areas and sent them to MOHME using a pre-structured excel format that allowed integration of the data (Fig. 1). The forms and the excel files included information about specialists’ name, surname, father’s name, national ID code, medical council ID code, socio-demographic characteristics, recruitment and job status, and specifications of the employing hospitals and affiliating organization. The data were collected from each hospital for all specialists who worked in that hospital in 2016, full-time or part-time, temporarily or permanently. The hospitals were given 1 month to collect the data. For hospitals with no reply, a reminder at the end of month 1 and another reminder at the end of month 2 were sent. A combination of criteria from each individual specialist was used to merge the data at the province and national level and to identify whether a specialist was involved in DP. In the first step, the individual specialists’ medical council ID code was used to match and merge the data. If this ID code was not available, the national ID code was used; eventually, if none of them was available for an individual, a combination of the first name, surname, and father’s name or their initials were used. These procedures were performed by SQL functions of Access database (Table 1).

**Table 1:** Reference banks and their data items for completion of collected data

Reference banks		Data items in the reference bank
Medical Council		Medical council code, type of specialty, sex, age
MOHME human resource management office		Faculty membership, experience, main occupation location
MOHME hospital management office		Full time status and experience
Medical Council Office permit/license		Office status
List of clinics in Iran		Name of clinic and its affiliation

### Data quality control

After receiving information from each hospital, via medical universities, the following steps were performed.

1. Preliminary assessment of data accuracy: The completed forms and excel files could potentially have two types of errors: “Instructions Errors” spotting through corresponding data with the provided instruction form and “Reference Errors” detected by matching a number of fields with the reference data banks that were already available at MOHME in terms of authenticity and accuracy. In case of having either of these errors, submitted forms were returned to the hospitals for correction and/or completion.
2. To minimize possible errors and increase accuracy of data collection and data merging, a standard extraction, transformation, and loading (ETL) method was adopted and used [16]. Separate data banks were developed for the MOHME (the headquarter, schools/research centers, hospitals and clinics), private sector organizations (hospitals, clinics, and physician offices), and other public sector organizations (hospitals and clinics). During transformation, the generated data banks entered a cycle of standardizing, cleaning, and completing.
  Standardizing: Based on the assessment, errors of the data in the Excel software were detected and corrected. They include misspellings, heterogeneity in naming, and heterogeneity in structure of the information.
  Cleaning: Accuracy and precision of the data was confirmed using recaptured data in data matching for each standardized data item with the reference banks.
  Completing: In this part, incomplete items consisting of faculty status, type of cooperation, demographic characteristics, etc. were completed through matching the data banks if needed. Also, one of the main objectives of this stage was completion of medical council codes for all records to proceed to the next stage which was identification of duplicate data between different extracted databases [18].

Finally, in the loading stage, required information for the study objective was extracted and refined from different sources and then loaded into one main concentrated data bank.

### Extraction of physicians with dual practice

Noting the nature of dual practice and attendance of physicians in more than one service delivery location, to identify these types of physicians, a data matching model was applied and therefore duplicate data of medical council codes were detected among the health ministry banks and other public and private banks.

After identifying duplicate data (indicating physicians with dual practice), their main occupation location were specified based on their type of recruitment relation listed in the forms. Afterwards, share of dual practice among public sector specialists in each province and its relation with other characteristics of the physicians and conditions of provinces were determined based on dual practice definition made by this study considering DP as employment of public sector physicians in the private sector and other dissimilar public sectors in terms of ownership.

### Analysis of the results

Logistic regression analysis was used to examine the relationships between DP and its contributory factors.

## Results

### Status of the study specialists

A total of 48 345 records were collected for 30 273 specialists from 858 (93%) hospitals out of total 925 hospitals from which 24 414 specialists were considered based on the definition of this study. The mean age of specialists was 46.23 ± 9.33 and (63% of them were male, 68% of specialists were employed in medical university hospitals, 69% of all specialists were non-faculty members, and 26% of all specialists were employed on a contract basis (Table 2).

**Table 2:** Demographic characteristics, university faculty membership, recruitment relation, and main occupation location of the specialists

Variables	#Specialists	%Specialists
Sex		
Male	15 356	63.2
Female	8 937	36.8
Age groups		
40>	6 690	27.4
40–45	4 185	17.1
45–55	9 699	39.7
55–65	2 792	11.4
65<	790	3.2
Main occupation location		
University hospital	20 668	84.7
Social Security hospital	2003	8.2
Armed forces hospital	551	2.3
Petrochemical Company hospital	240	1.0
Other public hospital	533	2.2
MOHME headquarter	370	1.5
Research center	20	0.1
Healthcare center	29	0.1
Faculty membership status		
Faculty member	7 565	31.0
Non-faculty member	16 849	69.0
Full-time employment status		
FTG*	8 826	36.15
Non-full-time	15 588	63.85
Recruitment relation		
Permanent	6 337	26.0
Peymani (semi-permanent)	2 644	10.8
Contractual	6 317	25.9
Zarib K	5 082	20.8
Payam avar	20	0.1
Other	2 868	11.7
Unspecified	1 146	4.7

^*^Full-time geographic (FTG) physicians are the ones who are not allowed to be active in any other locations/sectors except their main occupation location

**Fig. 2:**
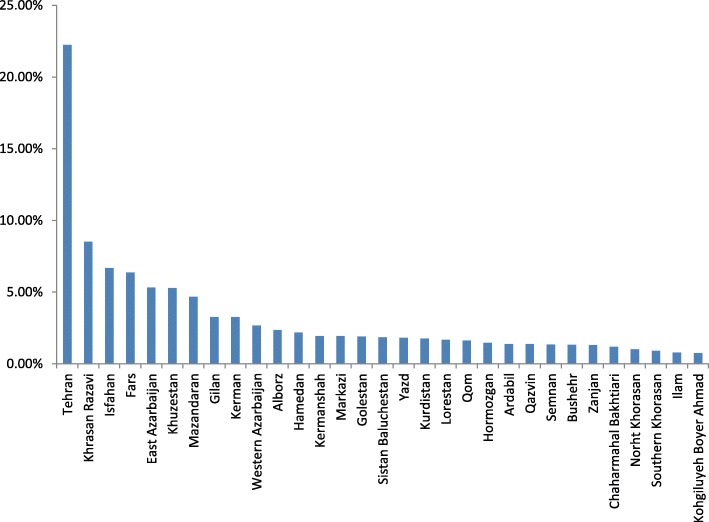
Distribution of physicians in the provinces of Iran

22.23% of specialists were in Tehran (Fig. 2). Kohgiluyeh-Boyer Ahmad, Ilam, and South Khorasan each had less than 1% of specialists.

### Dual practice status

A total of 11 638 (48%) public sector specialists were engaged in dual practice from which 9575 were in service through MOHME and 62% of non-full-time specialists had multiple job holdings (Table 3). Of 16 849 non-faculty member specialists, 29% were academic 48.60% were non-academic and had multiple job holdings (Table 3). The highest rate of DP occurred in the provinces of Qazvin (72%), Kohgiluyeh-Boyer Ahmad (70%), Gilan (69%), Alborz (57%), and East Azerbaijan (59%); compared to the provinces of Ilam, Hormozgan, Chaharmahal, and Bakhtiari, West Azarbaijan, Sistan, and Baluchestan had the lowest DP rates.

**Table 3:** Dual practice status of specialists by faculty membership status and full-time recruitment

	Total	
	#	%
Affiliated organization		
MOHME	9 575	45.40
Other public organs	2063	62.00
Faculty membership		
Faculty member (academic)	3 450	45.60
Non-faculty member	8 188	48.60
Full-time status		
Non-full-time	9 594	61.55
FTG	2044	23.16
Total dual practice	11 638	47.70

**Fig. 3:**
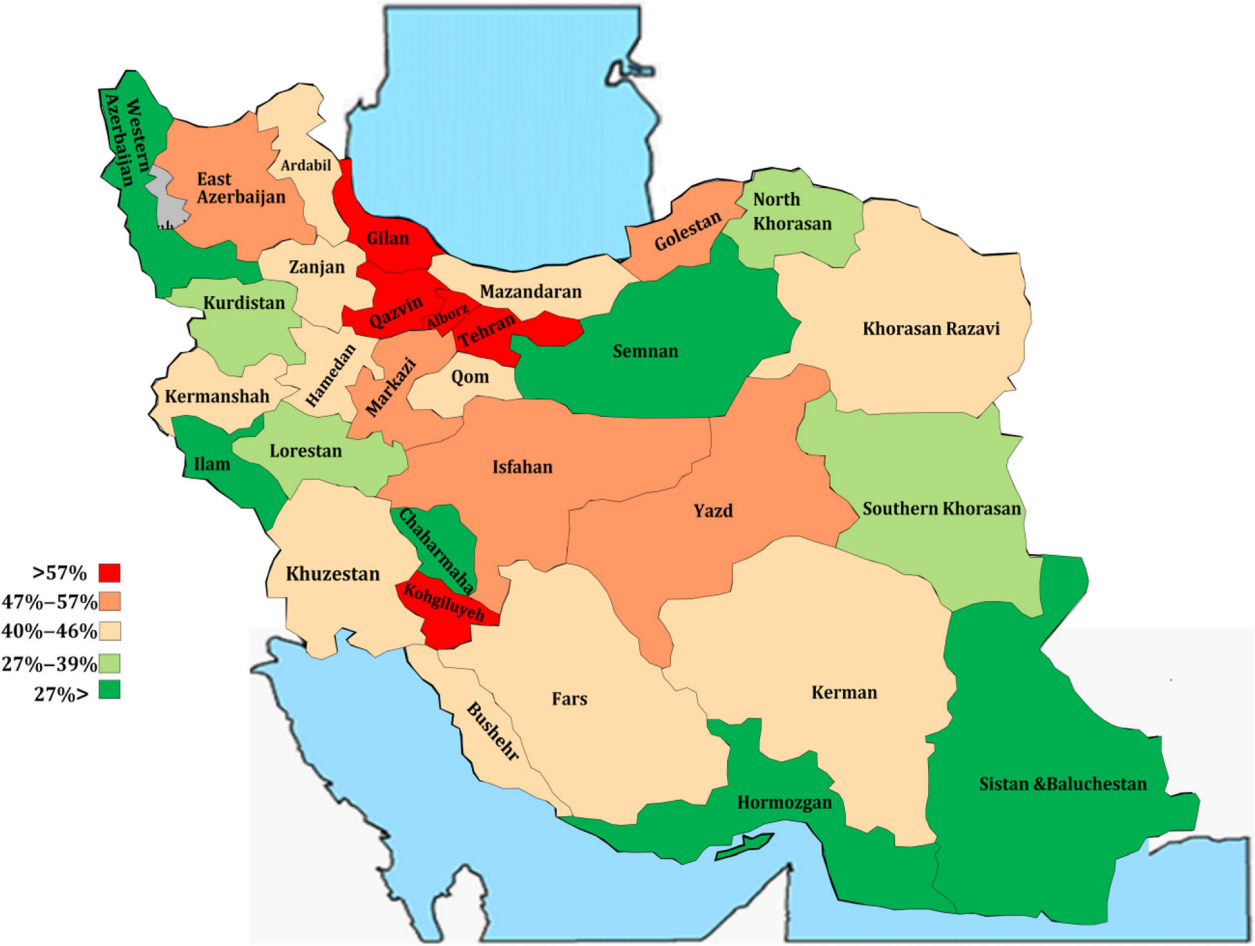
Map of geographical distribution of Iran’s specialists engaged in dual practice

Based on this geographic distribution map which illustrates DP distribution in five groups, provinces at the national borderline and the deprived provinces had a lower rate of DP compared to the provinces in the center or advantaged areas (Fig. 3).

### Factors contributing to dual practice

Female specialists had less chance of dual practice engagement with the private sector (odds ratio 0.76, *p* < 0.05). Similarly, faculty physicians to non-faculty ones (OR = 0.65) and full-time geographic physicians to non-full-time physicians had less chance of DP engagement (OR = 0.15). Specialists with the age 40 years and more, individuals with more than 5 years of job experience, and physicians with permanent public sector employment relation had also more DP (*p* < 0.05).

The total ratio of specialist per 10 000 population showed no significant correlation with DP (*p* > 0.05). However, there was a significant increase in DP with the increase of population. Specialists’ DP also increased (1.01 times) when adding one public hospital and also adding one private hospital. In addition, there was significant association between the share of private hospitals (proportion of private hospital to the total hospitals) with DP (OR = 1.6, *P* = 0.0001). There was also an inverse correlation between regions’ deprivation and DP. Each unit of reduction in regional deprivation is correlated with 2.05 times less DP.

## Discussion

In the present study, we used data matching to identify dual practitioners in a data bank which is created through our national survey while some studies used linking data between current registers [8, 21]. The difference of our study with the mentioned studies is that we had to create a cross-sectional data bank to study DP in the first place as in most developing countries there are no routinely registered data for the activities of physicians [10]. Furthermore, to our knowledge, this study of 24 293 specialists in Iran is one of the largest to investigate DP engagement in all regions of the country (Table 4). Some other studies were done through sampling and questionnaire [4, 8–10]. However, to consider different countries, based on objective of the study, two response biases might occur including, first, having a small sample of dual practitioners in the countries, especially in regions with small private sectors and, second, avoiding the truth presented by physicians in a self-reporting method where DP is banned. However, in Iran and similar countries where DP is legally banned, using the self-reporting method may result in information bias. Unlike some other studies which used the self-reporting method [4, 10], this study collected data from employers rather than employees.

**Table 4:** Correlation between specialists’ characteristics and the province features on dual practice engagement of public sector specialists

	Odds ratio (OR)	95% confidence interval		*P* level	*P* variable
		Lower limit	Upper limit		
Physician characteristics					
Sex					
Male	1				
Female	0.756	0.715	0.8	0.022	0.001
Age					
<40	1				
40–45	1.196	1.099	1.301	0.001	0.001
45–55	2.116	1.966	2.277	0.001	
55–65	5.651	5.065	6.304	0.001	
65<	3.176	2.735	3.688	0.001	
Experience					
≤ 5	1				0.001
6–14	1.359	1.249	1.479	0.001	
15–25	5.645	5.071	6.284	0.001	
25<	4.033	3.504	4.643	0.001	
Employment status					
Permanent	1				
Zarib K	0.218	0.197	0.241	0.001	0.001
Payam avar	0.051	0.007	0.386	0.004	
Peymani (semi-permanent)	0.431	0.391	0.476	0.001	
Contractual	1.035	0.961	1.115	0.362	
Others	0.779	0.709	0.856	0.001	
Unspecified	1.730	1.502	1.992	0.001	
Faculty membership status					
Non-faculty	1				
Faculty	0.647	0.607	0.690	0.001	0.001
Full-time status					
Non-FTG	1				
FTG	0.146	0.136	0.157	0.001	0.001
Provincial characteristics					
Total specialists (per 10 000 population)					
≤ 2.5	1				
2.5–4	1.795	0.919	3.505	0.087	0.091
4–5	1.957	0.834	4.593	0.123	
5≥	5.038	1.341	18.933	0.017	
Population					
≤ 500 000	1				
500 000–2 000 000	1.589	1.225	2.061	0.001	0.001
2 000 000–5 000 000	2.201	0.978	4.957	0.057	
5 000 000<	2.670	1.022	6.975	0.045	
Extent of regional deprivation*	2.053	1.498	2.813	0.001	0.001
Number of hospitals	1.008	1.001	1.016	0.031	0.031
Number of private hospitals	1.009	1.001	1.017	0.021	0.021
Share of private hospitals**	1.590	1.341	1.886	0.001	0.001

^*^Regional deprivation: the regional with lowest deprivation rate. Deprivation rate is ranked by the level of deprivation that showed with numbers 3 to 5. Depending on the region’s conditions, the most deprived region has the lowest rate (coefficient of deprivation of service points for doctors and paramedics. Tehran: Ministry of Health and Medical Education 2002 2002. Report No.: Contract No.: 24243)

^**^Share of private hospitals: proportion of private hospitals to the total hospitals in a province

Findings show that 11 638 (47.7%) specialists in Iran engage in Dual practice which is significantly lower than Austria (100%), Ireland (90%), the UK (63%), Australia (65.7%), Bangladesh (80%), Indonesia (70%), and Egypt (71%) [1, 14, 19, 20, 22]. This wide variation between the countries may be due to each country’s specific situations including number of workforce and supply [8, 21, 23]. There are currently two approaches for control of DP in Iran; complete ban for FTG specialists and partial restrictions using different incentives for non-full-time specialists [18]. We also presented our results for these two categories. We found that 60% of non FTG specialists engage in DP despite implementing incentive mechanisms. This shows that using the same strategy, there is still more DP in Iran compared to New Zealand (38%), Portugal (43%), and Denmark (37%) [4, 8, 9]. Moreover, in FTG specialists, despite complete ban, 24% of them are engaged in DP. This is similar to China where with complete ban on DP, 29.6% of physicians are engaged in DP due to increase of income [4]. The same situation is reported from Mozambique where 21% of specialists are engaged in DP [24].This shows that even complete ban has not routinely eliminated DP in many countries. The main factor affecting DP despite the banning strategies is financial influences. Higher payments by the private sector along with the government’s inability to pay specialists comparing to the private sector can lead to ignoring restrictive rules of DP [25–27]. In addition, inflexibility of the rules and mechanisms of control may increase the tendency of doctors towards the private sector [12, 28, 29].

We found that DP is reduced in deprived areas, regions with less population, areas with lower number of hospitals, and proportion of private hospitals (*p* < 0.05). This study showed that deprived provinces have lower level of DP and Tehran (capital) has a high level of DP. In Africa, regions with more facilities (2.2 per 10 000 population) had the most DP engagement (0.7 per 10 000 population). Moreover, it reported that DP is more frequent in areas with more service delivery units, more inpatient facilities, more operation capacities, more private hospital, and higher income [6]. McPake also found that DP is more frequent in areas with higher population, higher human development index, and areas with more physicians [7]. Findings of the present study are consistent with the mentioned findings. Probably one of the reasons for less prevalence of DP in non-urban and deprived areas is the poor development of the private sector and shortage of DP opportunities; therefore, the public sector remains the main provider of services. As studies have shown in areas where the private sector has better facilities, the chance for professionals to work simultaneously in the private sector will be greater [30–32]. In addition, other reasons especially in some developing countries include strategies such as post-graduate compulsory commitments [33] similar to Zarib K specialists in Iran who have mandatory commitments to work in deprived areas [18], and enforcing it through approving regulations in which they are not legally allowed through private practice [28]. Since these strategies have been developed in response to the shortage of specialists in these areas, they can affect DP prevalence in deprived areas as for the context of differences in the level of DP between urban and rural areas. Factors such as age, gender, social status, and professional characteristics of health workers have been also discussed; however, it seems they are not considered for policy making as of yet [2].

We found that female specialists were less likely to be engaged in dual practice in the private sector. In Norway, DP level among men and women were 25.0% and 14.2% respectively [8]. Socha and Bech also reported 2.1 times more DP in men compared with women [34]. McPake also found that men are more likely to have dual practice [35]. One of the reasons for this difference could be due to variation of marginal utility of income that is higher for males [36]. It means that the change in human satisfaction resulting from an increase or decrease in an individual’s income is higher among men. Therefore, men tend to have more income and the private sector is one way for them to increase their income [37]. Creating a balance between work and life is a decisive factor for women’s working hours and their DP. For example, having a new child or having a large family size showed a prominent negative impact on female’s DP [21]. Specialists with higher age and job experience had also more DP which is in line with other studies [18, 38]. Chawla has shown that aging and increase in expertise, skills, and reputation cause more attractiveness for the private sector to secure higher payments to physicians [39]. It is argued that elder physicians who had already established a reputation in the public sector engage more in dual practice. In Iran, young physicians with diplomas have mandatory commitments to work in the public sector and it is named as Zarib K full-time physicians who are subject to Act for Physicians & Paramedics Services [2]; on the other hand, since young physicians have fewer years of job experience and consequently they are less known/branded, they have less capacity of presence in the private market competing with senior physicians; therefore, they normally have lower level DP.

The present study is one of very limited number of studies which used a comprehensive approach to collect and analyze data for specialists from all Iran hospitals to explore the level of DP and its contributory factors.

## Conclusions

This study found that despite implementation of financial and non-financial incentives and facilities to retain specialists in the public sector in form of full-time or even with the existence of complete restriction laws, a significant proportion of physicians still engage in DP. With the current social demand and increased confidence of people on the private sector, this phenomenon seems inevitable. It appears that retention of specialists in public hospitals might not be feasible solely through enforcement of regulations, without considering other factors including the competition of the private sector in attracting experienced and senior physicians of the public sector. Considering a complete ban on DP might lead to movement of senior and experienced physicians from the public sector and drainage of these centers from experienced specialists. Enforcement of regulations that restrict DP might be more feasible than regulations with complete ban. Moreover, a multi-approach strategy is needed to control DP. This might include tax regulations, income cap, and limitation in work hours and number of patients visited/admitted in the private sector. In addition, we recommend the method we applied since, to our knowledge, it can alleviate research limitations in developing countries where there is no routine data registers, through a robust way of data gathering by creating a data bank and its indirect approach to address DP is specifically useful for the countries where DP is banned.

## Abbreviations

DP: Dual practice
ETL: Extraction, transformation, and loading
FTG: Full-time geographic
MOHME: Ministry of Health and Medical Education
MoP: Ministry of Petroleum
SSO: Social Security Organization
